# Improving Outcomes in Patients with CRC: The Role of Patient Reported Outcomes—An ESDO Report

**DOI:** 10.3390/cancers9060059

**Published:** 2017-05-26

**Authors:** Eric Van Cutsem, Aimery De Gramont, Geoffrey Henning, Philippe Rougier, Franck Bonnetain, Thomas Seufferlein

**Affiliations:** 1 Department of Digestive Oncology, University Hospitals Gasthuisberg, and KU Leuven, 493000 Leuven, Belgium; 2 Service d’Oncologie Médicale/Oncology Department, Institut hospitalier Franco-Britannique/Franco-British Institute, 92300 Levallois-Perret, France; aimerydegramont@gmail.com; 3 EuropaColon, London N16 8ST, UK; geoffrey@europacolon.com; 4 Hepato-Gastroenterology and Digestive Oncology Department, University Hospital Hotel Dieu, IMAD, 44000 Nantes, France; rougier.philippe2012@gmail.com; 5 Methodology and Quality of Life Department, University Hospital of Besançon & INSERM 1098, 25030 Jean Minjoz, France; franck.bonnetain@univ-fcomte.fr; 6 Department of Internal Medicine, University Hospital Ulm, 89081 Ulm, Germany; Thomas.Seufferlein@uniklinik-ulm.de

**Keywords:** metastatic colorectal cancer, patient-reported outcomes, health-related quality of life, registries, endpoints

## Abstract

Colorectal cancer is one of the most commonly diagnosed cancers worldwide and nearly half of patients will develop metastatic disease at some point during the course of their disease. The goal of anticancer therapy in this context is to extend survival, while trying to maximise the patient’s health-related quality of life. To this end, we need to understand how to incorporate patient-reported outcomes into clinical trials and routine practice to accurately assess if treatment strategies are providing clinical benefit for the patient. This review reflects the proceedings of a 2016 European Society of Digestive Oncology workshop, where the authors discussed the use of patient-reported outcomes to measure health-related quality of life when evaluating treatment during the management of colorectal cancer. A summary of the challenges associated with implementing patient-reported outcomes in clinical trials is provided, as well as a review of the current clinical evidence surrounding patient-reported outcomes in metastatic colorectal cancer.

## 1. Introduction

This article examines topics that were discussed by the authors at a European Society of Digestive Oncology (ESDO) workshop convened at the 2016 World Congress on Gastrointestinal Cancer in Barcelona, Spain. The objectives of the workshop were to highlight the importance of health-related quality of life (HRQoL) to patients with colorectal cancer (CRC) and its impact on treatment evaluation and decisions, and to evaluate the challenges of using patient-reported outcomes (PROs)—including HRQoL—and particularly how they can be standardized and used to improve patient management.

### 1.1. Epidemiology of Colorectal Cancer

Worldwide, CRC is one of the most frequently diagnosed cancers and one of the most common causes of cancer-related death [1]. In 2012, the estimated number of deaths from CRC in Europe was approximately 215,000, representing 12.2% of all deaths from cancer [1]. The worldwide incidence of CRC is high; it is the second most common cancer in women (614,000 cases, 9.2% of the total) and the third most common cancer in men (746,000 cases, 10.1% of the total). However, more men than women are diagnosed with and die from CRC each year in Europe [1].

The five-year prevalence of CRC (patients alive five years after diagnosis) in Europe in 2012 was 1.2 million, demonstrating that a large number of patients survive with the disease. The Eurocare-5 study reported five-year survival rates of 57% for colon cancer and 56% for rectal cancer [2]. Five-year survival rates are related to the stage of CRC at diagnosis. The survival rate is approximately 90% for local disease (stages I–II), 50% for CRC that has spread to the lymph nodes (stage III) and 10–20% for metastatic disease (stage IV) [3]. Table 1 summarises the incidence, mortality and five-year prevalence of colorectal cancer worldwide and in Europe.

### 1.2. Metastatic Colorectal Cancer

Approximately 25% of patients with CRC have metastatic disease at initial diagnosis, and almost 50% will develop metastases during the course of their disease [4]. Although the optimal treatment strategy for patients with unresectable metastatic CRC (mCRC) is constantly developing, the goals of treatment—including prolongation of overall survival, improving tumour-related symptoms, stopping tumour progression (pending improvement of overall survival and/or HRQoL), and/or maintaining HRQoL—remain important [4,5].

Standard treatments for mCRC are mainly palliative, and are based on chemotherapy and biologically-targeted agents. While palliative treatment for mCRC aims to increase the duration and maintain or improve the quality of the patient’s remaining life, this can be difficult given the toxicity profiles of the treatments administered. When treatments for mCRC are palliative, HRQoL considerations are crucial to understanding the impact of cancer on the patient [6]. Loss of health due to the cancer and/or the consequences of the treatment may result in psychophysical, functional, and social impairment—all of these affect HRQoL [7]. Bowel symptoms induced by cancer and/or its treatment such as diarrhoea, faecal control and constipation, as well as fatigue and loss of appetite are very common in CRC [7]. Patients with advanced cancer exist in a unique medical context, facing mortality and considering treatment options that have significant potential for toxicity.

Adverse events during cancer treatment can have a negative effect on HRQoL, so optimal therapy involves a balance between efficacy and safety. The aim is to increase the patient’s lifespan by a continuum of care that uses all the available and suitable drugs while at the same time ensuring a good quality of life for the patient. This means using treatment strategies such as induction and de-escalation/maintenance strategies, drug holidays, and so on to ensure that overall survival is not compromised but that HRQoL is maximised. To this end, HRQoL and PROs should be considered as a clinical endpoint in mCRC clinical trials to demonstrate that new treatment strategies are providing clinical benefit for the patient, and ultimately to individualise treatment on the basis of HRQoL and PROs to achieve the paradigm of precision/personalised medicine [8].

## 2. The Role of PROs in Improving Patient Management

### 2.1. PROs and Their Role in Shared Decision Making

A PRO is defined as any report about a health condition and its treatment that comes directly from the patient using a self-reported measure, without interpretation of the patient’s response by a physician or anyone else [9]. PRO data are collected via standardised questionnaires designed to measure an explicit concept such as symptoms, functioning, or HRQoL [10]. PRO instruments can measure treatment benefits by capturing concepts related to how a patient feels or functions with respect to a health condition. The concepts, events, behaviours, or feelings measured by PRO instruments can be readily observed or verified (e.g., walking) or can be non-observable, known only to the patient and not easily verified (e.g., feeling depressed). Assessment of symptom improvement or pertinent function depends on the patient’s perception [9].

Table 2 summarises the benefits of PROs. They can help to articulate the life changes that a patient experiences following an initial diagnosis of cancer and provide a picture of the patient’s state of mind. The routine measurement of PROs in oncology clinical practice has the potential to improve cancer care planning, monitoring, and management of patients by promoting better communication and shared decision making by patients and healthcare providers [11]. Furthermore, PRO measures are being advocated for use in routine clinical cancer practice and for the early detection of patient distress [12,13].

### 2.2. The Role of PROs in Clinical Trials

Patient involvement is increasingly being recognised as a component of healthcare, and PROs that quantify patients’ feelings or functions are now being considered as important endpoints in cancer clinical trials [14]. Some data can only be obtained from the patient during treatment, including symptoms such as fatigue, headache, depression, anxiety or sleep disturbances. The frequency and severity of these symptoms, and the level of disability resulting from them, can be provided only by the patient. In addition, patients can report the impact of cancer on their daily life, as well as their perception of the disease [15]. Although PROs including HRQoL are now being recognised as direct measures of benefit to patients and as independent endpoints in cancer clinical trials, they are poorly reported in cancer trials or may even be excluded as measures [16]. For example, between 2008 and 2011, only 30% of Phase III breast cancer clinical trials included PRO measures as an endpoint [17].

The Effectiveness Guidance Document (EGD) from the Center for Medical Technology Policy (CMTP) provides recommendations for the appropriate inclusion of PRO measures in the design and implementation of prospective clinical comparative effectiveness research (CER) in adult oncology, including but not limited to registries, prospective observational studies, randomised controlled trials, and pragmatic clinical trials [18]. PROs should be incorporated into all CER clinical trials, including an assessment of HRQoL in Phase II, Phase III, and/or Phase IV trials. The PRO measures should have demonstrated validity, reliability, and sensitivity, and the measures recommended include the EORTC QLQ-C30, FACT, MDASI, PRO-CTCAE, and PROMIS. HRQoL should be monitored at baseline and at regular time points throughout the study. A plan for the statistical analysis of PROs is needed, targeting dimensions, defining minimal clinically important differences, controlling for alpha type one error, and including power calculations and analysis of missing data. Analyses and data reporting should be completed alongside other clinical outcomes according to the CONSORT PRO Extension. Standardised approaches should be used for longitudinal analyses of HRQoL.

### 2.3. Challenges Associated with the Use of PROs in Clinical Trials

Efforts to standardise the use of PROs in clinical trials are underway. Standards for the PROs to be measured, the content to be included in clinical trial protocols, and the reporting of clinical trial findings have recently been established [19,20,21,22,23,24,25,26]. However, guidelines for the analyses and interpretation of PRO endpoints are insufficient. As a result, comparing results across clinical trials can be difficult, thus hindering the application of these research findings [27]. For example, the analysis of longitudinal HRQoL measures can be seriously hampered by informative drop-out. In longitudinal studies of quality of life, participants can drop out of the study because of illness or death. In such situations, the drop-out process may depend on the quality of life being experienced, rather than being random; hence, the incomplete follow-up of patients is called informative drop-out. Drop-out can be considered informative if its probability is dependent on unobserved (current and future) measures, such as drop-outs due to tumour relapse or death. Models such as the pattern mixture approach or joint modelling of response measures (e.g., HRQoL) and times to events (e.g., relapse and death) can be used to correct for informative drop-out [28].

Two Phase III clinical trials investigating the effects of bevacizumab plus radiotherapy–temozolomide in patients with newly diagnosed glioblastoma provide an example of the problems associated with a lack of standardisation of PROs. The two trials used the same tools to assess HRQoL but used different statistical approaches, and they found different results [29,30]. The divergent results made assessing the clinical value of adding bevacizumab in these patients difficult. The Setting International Standards in Analyzing Patient-Reported Outcomes and Quality of Life Endpoints Data (SISAQOL) initiative that has recently been established aims to produce a suite of tools, guidance, and international consensus standards for the analysis of HRQoL and other PRO data in randomised trials in cancer [27].

## 3. Clinical Evidence of PROs and HRQoL in mCRC

The inclusion of a patient-centred endpoint in mCRC clinical trials to reflect how a patient feels, functions, or survives may help to show that new treatment strategies are providing clinical benefit for the patient and ultimately to individualise treatment on the basis of HRQoL and PROs to achieve the paradigm of precision/personalised medicine [8]. The ultimate goal of anticancer therapy is to improve the quality and/or quantity of survival for patients with cancer [31]; PROs and HRQoL endpoints will facilitate this.

For example, the CO.2O Phase III randomised trial of patients with *KRAS* wild-type, chemotherapy-refractory mCRC showed that the combination of cetuximab (a monoclonal antibody targeting epidermal growth factor receptor, EGFR) plus brivanib alaninate (a dual inhibitor of vascular endothelial growth factor receptor and fibroblast growth factor receptor tyrosine kinases) delayed disease progression compared with cetuximab plus placebo. However, this observation of delayed disease progression was not associated with a longer period of time before deterioration in HRQoL. In fact, the combination of the two drugs led to an accelerated deterioration of HRQoL compared with single-agent cetuximab. The researchers suggested that this could be a result of the increased toxicity of the combined therapy, with the side effects offsetting any potential HRQoL benefit of delayed disease progression [32].

The time until definitive deterioration (TUDD) in HRQoL score has been defined as a method of longitudinal analysis in oncology. This method allows patients’ data to be preserved for analysis even if some of their questionnaires are missing, and allows the production of clinically meaningful and readable results for clinicians. To investigate the applicability of this method to mCRC, the HRQoL longitudinal changes in mCRC patients treated with FOLFOX4 versus FOLFOX7-FOLFIRI were analysed using data from the MIROX randomised Phase III trial [33]. The results showed that the type of treatment did not significantly influence longitudinal TUDD for the main dimension of the QLQ-C30 scores, suggesting that switching from oxaliplatin to irinotecan in the treatment of resectable mCRC does not improve patients’ HRQoL. These HRQoL results support findings about the lack of a clinically and statistically significant difference between FOLFOX4 and FOLFOX7–FOLFIRI [33].

The CO.17 trial demonstrated that cetuximab improves overall and progression-free survival in patients with advanced chemotherapy-refractory CRC—particularly in patients with wild-type *KRAS* tumours. Additionally, patients with wild-type tumours who received cetuximab in addition to best supportive care experienced significantly less HRQoL deterioration and a longer time before clinically significant deterioration occurred compared with those who received best supportive care alone [34].

In two randomised Phase III trials, the addition of panitumumab (another anti-EGFR monoclonal antibody) to FOLFOX4 (first line) or to FOLFIRI (second line) significantly increased progression-free survival in patients with wild-type *KRAS* mCRC. However, analyses of the HRQoL assessments made during these studies showed no statistically significant or clinically meaningful overall differences in the change in HRQoL in patients treated with panitumumab–chemotherapy compared with those treated with chemotherapy alone [35].

## 4. HRQoL Scoring in Routine Practice

HRQoL scores have the potential to be used not only as an endpoint in clinical trials, but also in routine practice to improve patients’ health during treatment. The results of the direct improvement of quality of life (DIQOL) randomised controlled trial in colorectal cancer patients using a tailored pathway with quality of life diagnosis and therapy will provide more information on whether a specific quality of life pathway improves patients’ HRQoL during follow-up care of their disease [36]. This approach has already been shown to improve HRQoL in breast cancer patients [36].

Use of PROs in routine practice can confer clinical benefits. Among adults receiving outpatient chemotherapy for advanced solid tumours at a large specialty cancer centre, self-reporting of symptoms with automated clinician e-mail alerts resulted in better HRQoL, fewer emergency room visits, fewer hospitalizations, a longer duration of palliative chemotherapy, and superior overall and quality-adjusted survival [37]. In another study, routine assessment of cancer patients’ HRQoL was found to improve physician–patient communication, in addition to improving patients’ HRQoL and emotional functioning [38]. HRQoL—particularly domains assessing physical function and symptoms such as pain—is known to be an independent prognostic factor for overall survival of mCRC patients [39,40,41]. Social functioning (as measured with the EORTC QLQ-C30 tool) has also been shown to be an independent prognostic factor for survival in mCRC patients [42].

## 5. Summary and Conclusions

Capturing patients’ subjective experience or HRQoL is a key endpoint in clinical trials of cancer therapy, including trials of CRC, and PROs are tools for achieving this. Self-reported symptoms can also be used in routine practice to help guide the management of patients with CRC. PRO data—including HRQoL—from clinical trials can be used to inform clinical decision making, health policy, and reimbursement decisions [25]. The Center for Medical Technology Policy has made recommendations for the design and implementation of PRO measures in CER [18]. However, no guidelines yet exist for longitudinal analysis of HRQoL, making comparison between trials difficult [26]. More evidence is needed on how PROs can be used in clinical practice and the potential benefits to patients.

## Figures and Tables

**Table 1:** Incidence, mortality, and five-year prevalence of colorectal cancer worldwide and in Europe.

	Incidence	Mortality	5-Year Prevalence
Worldwide	1,360,602 (9.7%)	693,933 (8.5%)	3,543,582 (10.9%)
Europe	447136 (13.1%)	214,833 (12.2%)	1,203,943 (13.3%)

**Table 2:** Benefits of patient-reported outcomes in clinical practice.

Patient-reported outcomes (PROs) allow for early detection of distress in a patient
PROs provide a valuable opportunity for the patient to be heard
When PROs have been used in clinical practice, they have led to an increase in symptom-related actions taken by the oncologist
Obtaining PRO information prior to a patient’s visit does not seem to increase the consultation time
PROs allow for symptoms to be discussed more openly and frequently, in particular chronic and non-specific symptoms
